# Supplementary material for: L-GIREMI uncovers RNA editing sites in long-read RNA-seq
Source: Genome Biol. 2023 Jul 20;24:171. doi: 10.1186/s13059-023-03012-w (PMC10360234; doi:10.1186/s13059-023-03012-w)
Supplement: Supplementary file 1 — Additional file 1: Fig. S1. Overview of the Alzheimer’s disease (AD) data. Fig. S2. Summary of mismatches observed in the AD dataset. Fig. S3. The data quality and RNA editing sites in the GM12878 long-read RNA-seq datasets generated by the Sequel II platform. Fig. S4. The data quality and RNA editing sites in the GM12878 long-read RNA-seq datasets generated by the Sequel platform. Fig. S5. Comparison of RNA editing sites identified in the short- and long-read data of GM12878. Fig. S6. Cumulative distribution of mutual information of pairs of REDIportal editing sites or pairs of SNPs in the same gene. Fig. S7. Histogram of the MI for the editing site. Fig. S8. Histograms of the read coverage of detected dsRNAs in two datasets. Fig. S9. Pattern of region-skipping and editing index of inverted Alu repeats. [file 13059_2023_3012_MOESM1_ESM.pdf]

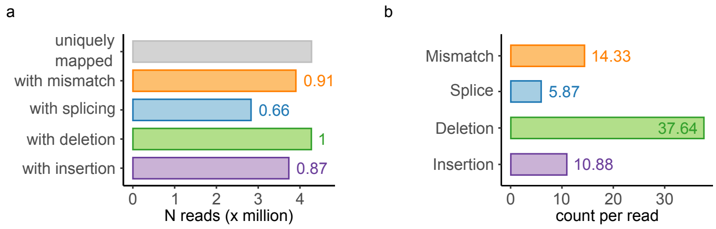

Figure S1: Overview of the AD dataset. (**a**) Number of reads with mismatches, insertions, deletions or spliced junctions. The value next to the bar is the fraction of reads among all uniquely mapped reads. (**b**) Average number of mismatches, insertions or deletions per read.

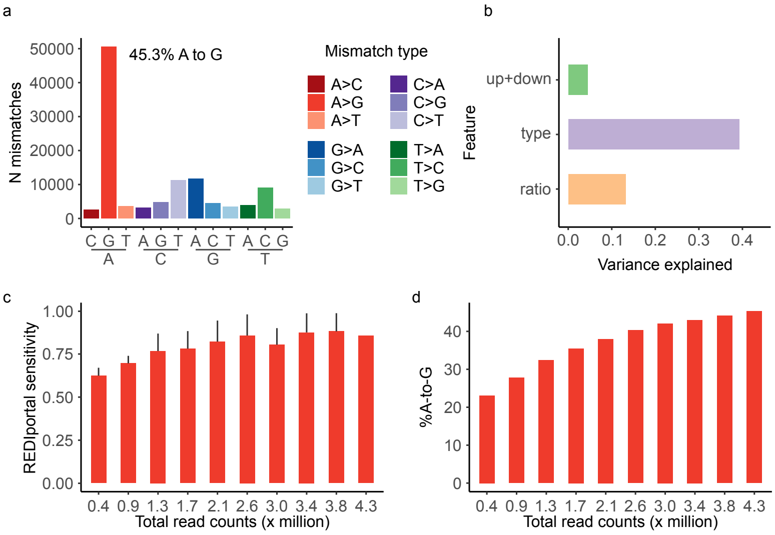

Figure S2: Summary of mismatches observed in the AD dataset. (**a**) Mismatches obtained in the AD dataset after the pre-filtering step (step 2, Figure 1) in L-GIREMI. (**b**) Variance explained by each feature (up+down: upstream and downstream nucleotides, ratio: allelic ratio, type: mismatch type). (**c**) Sensitivity (relative to identifiable REDIportal sites defined as those that passed the pre-filtering step and covered by at least 6 reads) of L-GIREMI given different read coverages (via random subsampling of the AD dataset). (**d**) %A-to-G among mismatches after the pre-filtering step in the subsets with different read coverages.

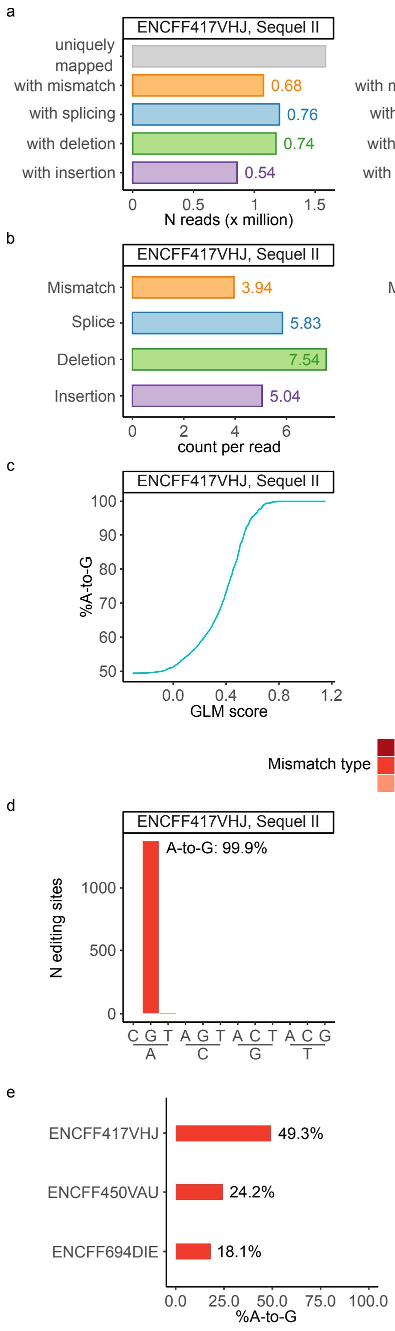

Figure S3: The data quality and RNA editing sites in the GM12878 long-read RNA-seq datasets generated by the Sequel II platform (ENCODE IDs: ENCFF417VHJ, ENCFF450VAU, ENCFF694DIE). (**a**) Number of reads with mismatches, insertions, deletions or spliced junctions. The value next to the bar is the fraction of reads among all uniquely mapped reads. (**b**) Average number of mismatches, insertions or deletions per read. (**c**) %A-to-G among all predicted editing sites vs. GLM scores. (**d**) RNA editing sites identified by L-GIREMI for the three datasets. The %A-to-G is shown in each graph. (**e**) %A-to-G among mismatches after the pre-filtering step (step 2, Figure 1) in L-GIREMI.

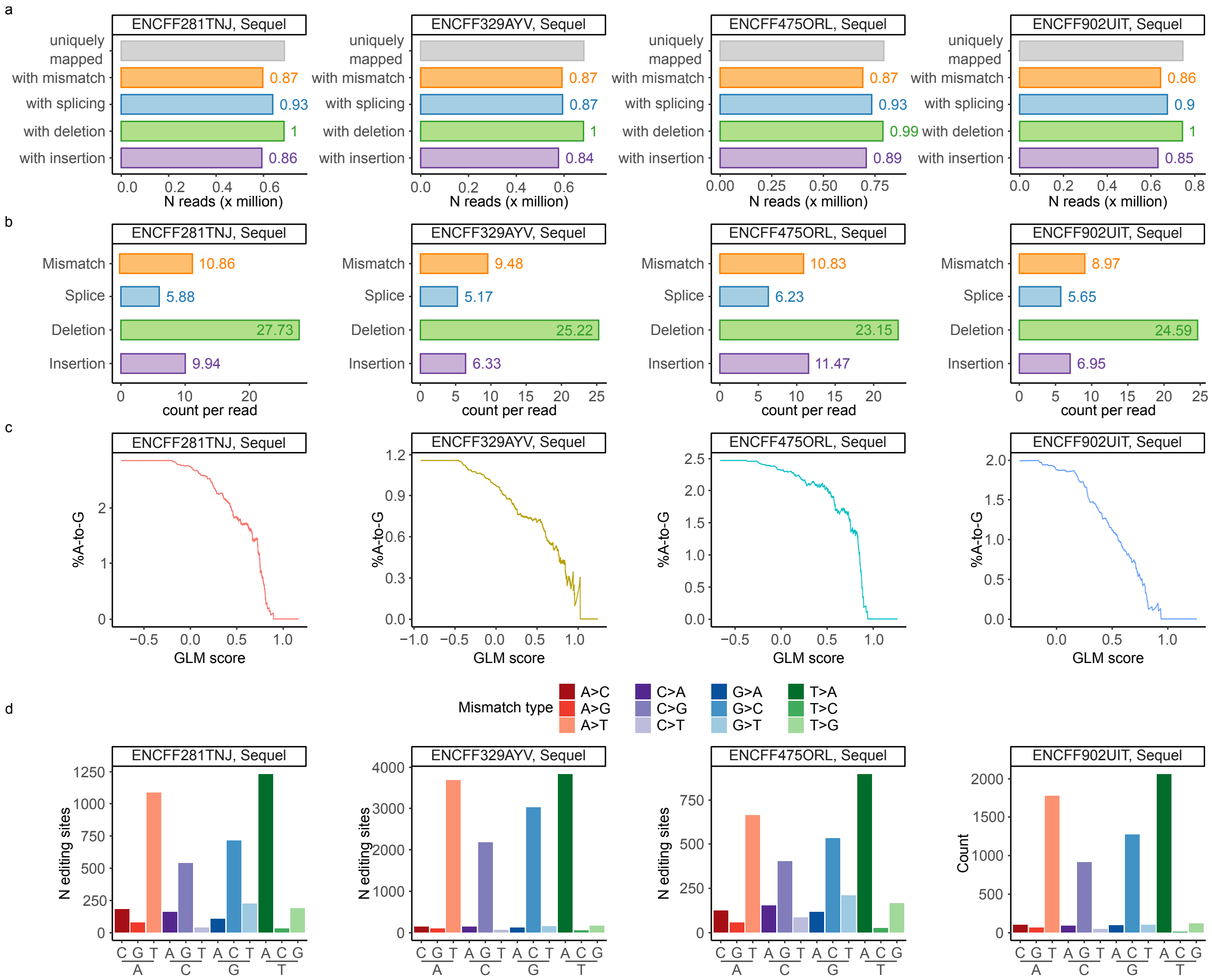

Figure S4: The data quality and RNA editing sites in the GM12878 long-read RNA-seq datasets generated by the Sequel platform (ENCODE IDs: ENCFF281TNJ, ENCFF475ORL, ENCFF329AYV, ENCFF902UIT). (**a-d**) Similar to Fig. S3 (**a-d**).

a

Repeat	Region	3ENCSR000COR, 30M Downsample		
		Counts	%A-to-G	%REDIportal
	Total	4391	86.15%	83.56%
Alu	All Alu region	3608	98.84%	97.67%
	Protein-coding gene 3' UTR	1701	99.24%	97.88%
	Protein-coding gene 5' UTR	35	100.00%	100.00%
	Protein-coding gene CDS	33	100.00%	100.00%
	Protein-coding gene intron	1311	98.86%	98.09%
	Non-coding gene exon	129	96.12%	96.12%
	Non-coding gene intron	143	98.60%	96.50%
	other	256	97.27%	94.92%
Non-Alu repeat	All non-Alu repeat region	137	60.58%	54.01%
	Protein-coding gene 3' UTR	26	61.54%	53.85%
	Protein-coding gene 5' UTR	2	50.00%	50.00%
	Protein-coding gene CDS	1	0.00%	0.00%
	Protein-coding gene intron	44	68.18%	63.64%
	Non-coding gene exon	18	44.44%	27.78%
	Non-coding gene intron	30	56.67%	53.33%
	other	16	68.75%	62.50%
not in repeats	All not in repeats	646	20.74%	10.99%
	Protein-coding gene 3' UTR	184	32.61%	22.83%
	Protein-coding gene 5' UTR	29	17.24%	3.45%
	Protein-coding gene CDS	246	13.01%	1.22%
	Protein-coding gene intron	71	26.76%	18.31%
	Non-coding gene exon	67	11.94%	7.46%
	Non-coding gene intron	21	0.00%	0.00%
	other	28	35.71%	25.00%

b

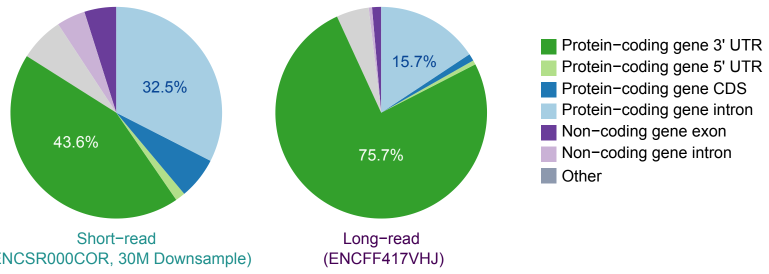

c

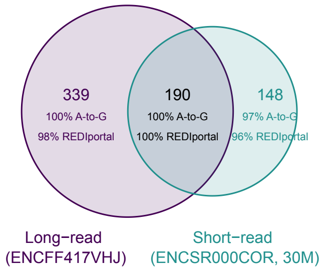

Figure S5: Comparison of RNA editing sites identified in the short- and long-read data of GM12878. (**a**) RNA editing sites identified in the short-read data (ENCSR000COR, 30M downsample). (**b**) Distribution of RNA editing sites in different types of regions. (**c**) Overlap of RNA editing sites identified in the short- and long-read data, among all testable RNA sites common to the two datasets.

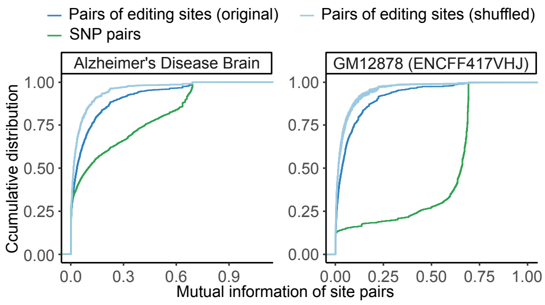

Figure S6: Cumulative distribution of mutual information of pairs of REDIportal editing sites or pairs of SNPs in the same gene. Compared to the shuffled controls, both editing sites and SNPs show higher levels of linkage ($p < 0.001$ for all comparisons, KS test) although the latter were associated with much higher mutual information.

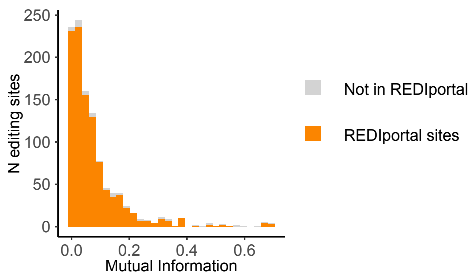

Figure S7: Histogram of the MI for the editing sites identified in the ENCFF417VHJ dataset. Orange for sites in REDIportal, and gray for sites not in REDIportal.

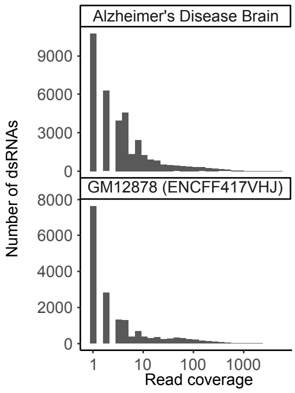

Figure S8: Histograms of the read coverage of detected dsRNAs (n read $\leq$ 1) in the AD (top) or GM12878 data (bottom).

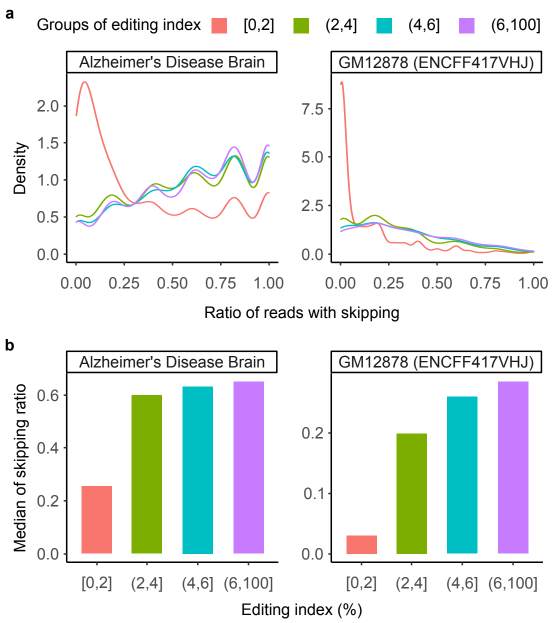

Figure S9: Pattern of region-skipping and editing index of inverted *Alu* repeats. The *Alu* repeats were separated into 4 groups according to their editing index. (**a**) Ratio of reads with skipping patterns. (**b**) Median of skipping ratio. For the AD dataset, the numbers of *Alu* pairs in each editing index group are (editing index low to high): 4135, 1492, 801 and 1130. For the GM12878 dataset, the numbers of *Alu* pairs in each group are: 3326, 318, 130 and 178.
