# Supplementary material for: L-GIREMI uncovers RNA editing sites in long-read RNA-seq
Source: Genome Biol. 2023 Jul 20;24:171. doi: 10.1186/s13059-023-03012-w (PMC10360234; doi:10.1186/s13059-023-03012-w)
Supplement: Supplementary file 3 — Additional file 3: Peer review history. [file 13059_2023_3012_MOESM3_ESM.docx]

**Review history**

**First round of review**

**Reviewer 1**

The authors present an extension of their GIREMI approach, applicable for RNA editing detection using long-reads with relatively high sequencing error rates. As far as I know this is the first attempt at editing detection using such long reads. Accordingly, it is highly timely and of much current interest. I thus consider it an important contribution that would be appreciated by the RNA editing community, and would like to recommend publication provided that the following comments are addressed.

Here are a few questions, comments and suggestions, in no particular order:

1. I think it is worth mentioning that the idea of using MI between mismatches (MMs) to classify them to SNPs vs. Editing Sites (ES), introduced by the same group in their 2015 paper, should work even better for long reads. With short reads, the MI cannot be computed for many isolated MMs (as indicated in Supp. Table 4 of the 2015 paper). With long reads, this problem should be solved in most cases.

2. However, there is still a concern that the method would work better for regions that are enriched in SNPs and ESs, and less well in other regions. In particular, classifying MMs in the coding sequence where ES tend to be more isolated and SNPs are less frequent could be more challenging. It would be helpful to use a high-quality set of previously-identified ES in the coding region as a positive control, and see how many of these sites (assuming they exhibit a MM in the given dataset) are identified correctly using the MI approach.

3. It is only natural to expect a comparison of the present method with the more traditional "genome-aware" methods, as you did in your 2015 paper.

4. It is well known that detecting editing in Alu repeats is quite easy (essentially all adenosines are edited) and more complex in the coding region. Please report in the above comparison (and throughout the paper) the number of sites and %AG for each region separately, using the same scheme applied in your 2015 paper (Supp. Tables 2-3).

5. The use of long reads allows for examining the effect of distance on the MI. Could you look at the MI distributions for difference distances between the sites? I think it is possible that you may improve the accuracy by focusing on pairs at some specific distance-regions. In particular, ES correlation decay quite quickly with distance (See Paz-Yaacov et al PNAS 2010; Duan et al MBE 2017; Moldovan et al, Sci. Rep. 2022), and it might be helpful to use for classification only MI values calculated for sites that are at least X bp apart. By the way, these works could be cited in p.6 l. 47, where you mention that "This question was challenging to address using short reads".

6. Fig 2b presents the MI distributions for pairs of known SNPs and pairs of "unknown" sites. What is the distribution for pairs containing one SNP and one "unknown" site?

7. Figure 3: the way I see it, the main purpose of GIREMI is to exclude SNPs from the detected putative ES list, without the need in matching gDNA-seq data. Accordingly, the interesting comparison to present is not the final set vs. the original unfiltered one (which, to a large extent, demonstrates the quality of the filters), but rather looking the %AG in the filtered data (as shown in Sup. Figure 2) compared to the same set after applying the MI cutoff, and then again to the results of the GLM.

8. P.4 lines 35-40. Please clarify: how many sites were classified based on MI alone, before employing the GLM (with the p<0.05 cutoff)? What was the %AG? Again, please distinct between coding, non-coding, Alu and non-Alu etc.

9. The study of "inequality" between reads using the Gini index is novel. Note however, that the Gini index measures the overall editing level (number of edits), and is not so sensitive to specific site-site correlations. I wish to refer to the above papers again, where it is shown that site-site correlations have two components. One which is virtually distance independent, likely representing the overall ADAR accessibility of the specific molecule. The other depends on the interplay between sites within the molecule, and is distance specific. Both contribute to "inequality". It would be nice to use your data to further clarify this issue which is of current importance in view of the efforts to use Site-Directed RNA Editing for RNA engineering.

10. The phenomenon of "skipped regions" in the vicinity of dsRNAs was reported a while ago (Osenberg et al, RNA 2009). You looked for dsRNA only using the proxy of extensive editing. It would be interesting to test whether putative dsRNAs (e.g. paired Alu regions) that are not extensively edited are also showing this behavior.

11. The MI formula takes into account all four possibilities per nucleotide (or rather, all 16 possibilities of pairs). However, this could inflate the MI artificially due to low-rate sequencing errors. I suggest you calculate the MI for each pair using only the two most frequent alleles at each site. By the way, I assume the log in the formula is the natural log. Please specify.

12. Regarding the GLM: the mismatch type is mentioned as one of the features used. That is, the model may have an intrinsic preference for A-to-G mismatches. I understand that the model "selects" this preference based on the learning set, but still this is not a good practice in my opinion. Please remove this feature and recalculate the %AG.

13. Please provide details regarding the relative importance of the different features in the GLM? How much worse are the results if the model does not take into account the neighboring nucleotides, or the allelic ratio, or the MI? What is the improvement in using the GLM over relying on the MI alone?

**Reviewer 2**

The Authors present a new methodology, called L-GIREMI, able to detect RNA editing events through PacBio long reads. This method is able to handle sequencing errors, typical of third generation sequencers, and to prioritise bona fide editing events as they do not show the significant linkage expected from allelic variants.
The proposed methodology, although not completely original (see https://doi.org/10.1093/bioinformatics/btx473) seems reasonable and effective and the conclusion adequately supported by the data.
However, I have two major issues that should be adequately addressed.
1) Most of editing events have a low rate affecting a small fraction of transcripts. The use of long reads, which generally provide a lower coverage with respect to short reads may be problematic in some cases. The Authors should provide an accurate evaluation of the sensitivity of the method with robust information on the correlation between observed coverage and detectable editing level.
2) As this methods is alternative to the use of short reads, the Authors should provide a comparative assessment by analysing the results obtained from both on the same samples.
Additionally, they could eventually discuss the use of combined information.

**Reviewer 3**

L-GIREMI is a new method to detect RNA-editing in the PacBio long-read RNA-seq data using the error profile. L-GIREMI can distinguish the RNA editing sites with mismatches arising from sequencing errors. 98.1% of RNA editing sites identified by the method are A-to-G (Figure 2a), compared to the small fraction of A-to-G of the raw mismatches (Figure 2c). The A-to-G fraction among the predicted RNA editing sites remains relatively high with lower read coverage, although the number of predicted sites decreased. It shows the accuracy of their method is robust for a wide range of total read coverage. In addition to method development, the authors also used the single molecule information plus long read length to test whether the RNA-editing events will co-occur in the same RNA molecule and to conduct the allele-specific RNA editing events analysis. They found a region-skipping pattern of long reads (Figure 5) in the vicinity of RNA editing sites. Below are a few comments to this version of the manuscript:

1. Similar to the authors' previous method GIREMI (Zhang & Xiao,2015), L-GIREMI utilized mutual information to predict RNA editing sites in the first step, given the assumption that most RNA editing events are expected to occur independently of the allelic origin of the mRNA. These predicted RNA editing sites are then used as the training data for the generalized linear model. I wonder if other training data, such as gold standard by experiments, is possible/available to improve the accuracy of prediction. If it is technically hard to generate such data, but a discussion could be added to explain the limitation of training and possible gaps and improvement.
2. To further demonstrate L-GIREMI's performance, comparison with other software, including the short-read ones or experimental validation should be added.
3. In addition to the evaluation metric "A-to-G fraction of RNA editing sites", more statistics should be added to evaluate the performance.

**Authors’ response to reviewers**

We thank all reviewers for their very valuable comments and constructive suggestions, which have helped strengthen our manuscript. We have revised the manuscript, with the main changes highlighted in green font. Below we provide a detailed point by point response.

# Reviewer #1:

The authors present an extension of their GIREMI approach, applicable for RNA editing detection using long-reads with relatively high sequencing error rates. As far as I know this is the first attempt at editing detection using such long reads. Accordingly, it is highly timely and of much current interest. I thus consider it an important contribution that would be appreciated by the RNA editing community, and would like to recommend publication provided that the following comments are addressed.

Here are a few questions, comments and suggestions, in no particular order:

1. I think it is worth mentioning that the idea of using MI between mismatches (MMs) to classify them to SNPs vs. Editing Sites (ES), introduced by the same group in their 2015 paper, should work even better for long reads. With short reads, the MI cannot be computed for many isolated MMs (as indicated in Supp. Table 4 of the 2015 paper). With long reads, this problem should be solved in most cases.

Thank you for this great point. We have added a discussion about this point (page 10). In theory, long reads afford an advantage in using MI to classify SNPs and ES. However, a practical challenge exists due to the relatively high level of sequencing errors in the current third-generation sequencing reads. These sequencing errors (also expected to have low MI relative to SNPs, similarly as editing sites) may lead to false positives in the MI-predicted editing sites (details below). The GLM step is necessary to further enhance the performance of the method. Nonetheless, with future improvements in sequencing technologies, the MI method will become increasingly effective. When the sequencing error rate of long reads reaches the same level as in short reads, we expect the %AG resulting from MI will be as high as that observed in the MI results of short reads (around 99%). Thus, in that case, we expect MI alone may be sufficient and GLM may not be needed for RNA editing identification (which of course needs a detailed evaluation in the future).

2. However, there is still a concern that the method would work better for regions that are enriched in SNPs and ESs, and less well in other regions. In particular, classifying MMs in the coding sequence where ES tend to be more isolated and SNPs are less frequent could be more challenging. It would be helpful to use a high-quality set of previously-identified ES in the coding region as a positive control, and see how many of these sites (assuming they exhibit a MM in the given dataset) are identified correctly using the MI approach.

We appreciate this suggestion. We attempted to use the GM12878 data for this analysis as its genome is well-annotated and previous work has identified ES for this cell line.

However, due to the relatively shallow coverage in the long-read data, only a small number of ES were present with adequate read coverage in the coding region. For example, only 10 coding ES identified in the GM12878 short-read RNA-seq were covered with at least 6 reads, 4 of which passed the MI test, 6 of which were identified by L-GIREMI. The numbers here are too low to enable a method evaluation.

Instead, we used the Alzheimer’s disease (AD) dataset and examined its coding ES. Since the genome info is not available for this sample, we examined the %AG and % REDIportal among the predicted coding ES. In total, L-GIREMI identified 501 coding ES, of which 163 were identified by the MI step. Among the MI-identified coding ES, 74% (121) were A-to-G sites, and 49% (80) were annotated in the REDIportal database. Among the L-GIREMI-identified (MI+GLM) coding ES, 72% (359) were A-to-G sites, and 37% (185) were annotated in the REDIPotal database.

To show a more comprehensive comparison and based on the comments below, we’ve included Table S1 and S2 to show the results of L-GIREMI and MI-alone in different types of regions for the AD and GM12878 dataset, together with results by the genome- aware method (for comment #3 below). Based on these data, L-GIREMI outperformed the genome-aware method for both datasets.

3. It is only natural to expect a comparison of the present method with the more traditional "genome-aware" methods, as you did in your 2015 paper.

We thank the reviewer for this suggestion. We carried out the “genome-aware” analysis for the long-read GM12878 data using the genomic SNPs of this cell line (from GIAB version 4.22.1 annotation). The same pre-filtering steps as applied for L-GIREMI were used here to enable a fair comparison. To compare the performance of the two methods, we calculated the %AG in the predicted ES, and the % of predicted ES that are REDIportal sites. As shown in Fig. R1 below, L-GIREMI led to a high %AG (99.93%) and high %REDIportal (94.96%), whereas the “genome-aware” strategy had much lower percentages (38.88% AG and 39.74% REDIportal). Thus, L-GIREMI outperforms the “genome-aware” strategy based on these data. Please see the response to comment #4 below for a more detailed comparison.

Undoubtedly, the “genome-aware” method was quite effective for short-reads data. However, its performance relies on the relatively high accuracy of the reads, that is, low sequencing error rates. For long reads, due to the existence of relatively abundant sequencing errors, the “genome-aware” method (plus the pre-filtering steps) does not appear to be adequate based on the above results. In contrast, sequencing errors can be mitigated (to certain degree) by the MI and GLM steps of L-GIREMI, as both steps improved the %AG and %REDIportal as shown in the response to comment #4 below.

We have revised the manuscript to include the detailed region-specific comparison to the “genome-aware” method for the GM12878 data (Table S2). In addition, for the AD data where the genome information is not available, we used dbSNPs to filter out likely genetic variants from the sites that passed the pre-filtering steps, which we call the

“pseudo-genome-aware” method, and included the region-specific comparison with L- GIREMI as Table S1. We have included a discussion of the limitations of the genome- aware method (page 10).

**Figure R1**. RNA editing sites predicted by the genome-aware and L-GIREMI methods, respectively, for GM12878 (ENCODE data ID: ENCFF417VHJ). Left %A-to-G; Right:%REDIportal.

4. It is well known that detecting editing in Alu repeats is quite easy (essentially all adenosines are edited) and more complex in the coding region. Please report in the above comparison (and throughout the paper) the number of sites and %AG for each region separately, using the same scheme applied in your 2015 paper (Supp. Tables 2- 3).

As mentioned above, we have included two supplementary tables (Tables S1 and S2) to show the number of sites, %AG, %REDIportal of L-GIREMI, compared to the genome-aware (for GM12878) or pseudo-genome-aware (for AD) methods. We separated the editing sites into different types of regions as suggested by the reviewer. In addition, we show the results for the different steps of our method (pre-filter only, pre- filter + MI, and L-GIREMI (i.e., pre-filter + MI + GLM)). L-GIREMI outperformed genome- aware methods in all types of regions.

5. The use of long reads allows for examining the effect of distance on the MI. Could you look at the MI distributions for difference distances between the sites? I think it is possible that you may improve the accuracy by focusing on pairs at some specific distance-regions. In particular, ES correlation decay quite quickly with distance (See Paz-Yaacov et al PNAS 2010; Duan et al MBE 2017; Moldovan et al, Sci. Rep. 2022), and it might be helpful to use for classification only MI values calculated for sites that are at least X bp apart. By the way, these works could be cited in p.6 l. 47, where you mention that "This question was challenging to address using short reads".

We appreciate this comment. As expected by the reviewer and consistent with the literature mentioned above, we observed that MI of pairs of ES decreased with

increasing distance between the pair (Fig. R2a). We have cited the papers mentioned here. However, we note that when identifying editing sites with MI, we used the MI value between a SNP and an unknown mismatch, rather than that between a pair of unknown mismatches. Thus, we also examined the MI between a SNP and an unknown mismatch for different distances (Fig. R2b). This MI is not dependent on distance. Thus, we did not use a distance cutoff in this MI calculation.

**Figure R2.** Median of mutual information (MI) of site pairs with different distance. (**a**) Median of MI for pairs of editing sites predicted by L-GIREMI. (**b**) Median of MI between SNPs and unknown mismatches.

6. Fig 2b presents the MI distributions for pairs of known SNPs and pairs of "unknown" sites. What is the distribution for pairs containing one SNP and one "unknown" site?

Indeed, in the original Fig. 2b, the MI for pairs containing one SNP and one unknown site was presented (originally labeled as non-dbSNP mismatch). We apologize for the confusion and have updated the legend of Fig. 2b to clarify. Based on this comment, we also calculated the MI for pairs of unknown sites. As shown in Fig. R3 below, the MI between pairs of unknown sites and that between SNP-unknown site are largely similarly, both being much lower than the MI of SNP pairs. In L-GIREMI, we used the MI between SNP-unknown site to predict editing sites (compared against the MI of SNP pairs).

**Figure R3.** Mutual information (MI) for pairs of putative heterozygous SNPs (based on dbSNP), non-dbSNP mismatches relative to putative SNPs, and pairs of non-dbSNP mismatches.

7. Figure 3: the way I see it, the main purpose of GIREMI is to exclude SNPs from the detected putative ES list, without the need in matching gDNA-seq data. Accordingly, the interesting comparison to present is not the final set vs. the original unfiltered one (which, to a large extent, demonstrates the quality of the filters), but rather looking

the %AG in the filtered data (as shown in Sup. Figure 2) compared to the same set after applying the MI cutoff, and then again to the results of the GLM.

This is a good point. We have now expanded the analysis to include comparison

of %AG in the pre-filtered data, after applying MI and after L-GIREMI (MI + GLM). As shown in Fig. R4 below, for both the AD and GM12878 dataset, L-GIREMI yielded the highest %AG, and sites passing the pre-filters had the lowest %AG. The MI step alone didn’t give the best %AG, presumably due to existence of sequencing errors in the long reads. Thus, the GLM step is necessary. Detailed results are included in the new Tables S1 and S2. In addition, we wish to clarify that the original Fig. S2 showed results after pre-filtering and an additional SNP filter (where dbSNPs were removed from the predicted editing sites, equivalent to the pseudo-genome-aware method). We apologize for the oversight, and have corrected Fig. S2 to include results only after pre-filtering.

**Figure R4.** %A-to-G of sites identified at different steps of the method: pre-filtering, MI, and L-GIREMI for the AD and GM12878 datasets, respectively.

8. P.4 lines 35-40. Please clarify: how many sites were classified based on MI alone, before employing the GLM (with the p<0.05 cutoff)? What was the %AG? Again, please distinct between coding, non-coding, Alu and non-Alu etc.

Please see response to comments #4, 7, with the detailed results shown in Table S1 and S2.

9. The study of "inequality" between reads using the Gini index is novel. Note however, that the Gini index measures the overall editing level (number of edits), and is not so sensitive to specific site-site correlations. I wish to refer to the above papers again, where it is shown that site-site correlations have two components. One which is virtually distance independent, likely representing the overall ADAR accessibility of the specific molecule. The other depends on the interplay between sites within the molecule, and is distance specific. Both contribute to "inequality". It would be nice to use your data to further clarify this issue which is of current importance in view of the efforts to use Site-Directed RNA Editing for RNA engineering.

We agree with the reviewer that the Gini index does not directly measure site-site correlations. A direct evaluation of site-site correlations had been presented in great detail by the publications cited by the reviewer using short reads data. With the long- read data, we calculated the same site-site correlation coefficient (r) as Moldovan et al, Sci. Rep. 2022. As shown in Fig. R5, the r values showed an overall dependence on the distance between the two sites, especially in the smaller distance range. This distance dependence, although consistent with Fig. 2A of Moldovan et al, is not as strong as observed in their work, possibly due to the lower coverage afforded by the long-read data than in the short-read data. Given this limitation of the current long read datasets and the fact that this question has been examined in detail using short-read data, we decided not to pursue this topic for now. Once deep coverage of long-read RNA-seq is a reality, it would be very interesting to revisit this question.

**Figure R5.** Correlation coefficients of ES pairs with respect to the distances between sites.

10. The phenomenon of "skipped regions" in the vicinity of dsRNAs was reported a while ago (Osenberg et al, RNA 2009). You looked for dsRNA only using the proxy of extensive editing. It would be interesting to test whether putative dsRNAs (e.g. paired Alu regions) that are not extensively edited are also showing this behavior.

Thanks for the interesting question. We have cited the Osenberg paper in the revised manuscript. As suggested, we took regions with inverted Alu repeats (within 1000nt) and separated them into 4 groups according to their editing index. As shown in Fig. R6, the group with the lowest editing index had the smallest skipping ratios, whereas groups with modest editing showed considerable levels of skipping. This observation indicates that Alu pairs with very low editing levels may not form strong dsRNA structures (assuming read-skipping occurs only in dsRNA regions). Therefore, using editing as a proxy of dsRNA structures is an effective approach. We have added a discussion of this point in the manuscript (page 11).

**Figure R6.** Pattern of region-skipping and editing index of inverted Alu repeats. The Alu repeats were separated into 4 groups according to their editing index. (**a**) Ratio of reads with skipping patterns. (**b**) Median of skipping ratio. For the AD dataset, the numbers of Alu pairs in each editing index group are (editing index low to high): 4135, 1492, 801 and 1130. For the GM12878 dataset, the numbers of Alu pairs in each group are: 3326, 318, 130 and 178.

11. The MI formula takes into account all four possibilities per nucleotide (or rather, all 16 possibilities of pairs). However, this could inflate the MI artificially due to low-rate sequencing errors. I suggest you calculate the MI for each pair using only the two most

frequent alleles at each site. By the way, I assume the log in the formula is the natural log. Please specify.

Indeed, the MI was calculated using only the two most frequent alleles for each pair. The log in the formula is the natural log. We apologize for the ambiguity in the original Methods description and have clarified these questions (page 13).

12. Regarding the GLM: the mismatch type is mentioned as one of the features used. That is, the model may have an intrinsic preference for A-to-G mismatches. I understand that the model "selects" this preference based on the learning set, but still this is not a good practice in my opinion. Please remove this feature and recalculate

the %AG.

We understand this concern. However, as the reviewer commented, since the mismatch type is a feature derived from the training dataset, no explicit bias was imposed by the user to favor A-to-G sites (or any other type of mismatches). Thus, we think it is an important feature to include, which should improve model performance if the training data is relatively accurate. As suggested by the reviewer, we removed this feature and recalculated the %AG. Without the mismatch type feature, the %AG is reduced to 75% for the AD data and 55% for the GM12878 data.

We think that including the mismatch type feature is preferred, as the feature is “learned” from the training data. This feature is especially important given data with relatively high levels of sequencing errors. If the training data possess a type of editing that is more prevalent than other types, we should leverage that information to improve performance. For example, suppose L-GIREMI is used on data derived from a species where a novel editing type other than A-to-I is prevalent, including this feature of mismatch type will greatly facilitate identification of the novel type of editing if the MI- derived training data possess enrichment of this type of mismatch. For this reason, we elected to keep this feature in the model.

13. Please provide details regarding the relative importance of the different features in the GLM? How much worse are the results if the model does not take into account the neighboring nucleotides, or the allelic ratio, or the MI? What is the improvement in using the GLM over relying on the MI alone?

We appreciate this question. Fig. R7a shows the variance explained by the neighboring nucleotides, the allelic ratio, or the mismatch type in the GLM (MI is not a feature in GLM). On the other hand, as shown in Fig. R7b, removing these features (other than the mismatch type) individually induced only small changes in %AG or %REDIportal, likely reflecting existence of redundancy among the features. The neighboring nucleotides and allelic ratio features do not seem to contribute substantially in the datasets tested here. We think the reason is that there exist sequencing errors in the MI-predicted editing sites that are used as training data for the GLM, and these features are especially susceptible to inaccuracy in the training data.

Regarding the improvement in using the GLM over relying on the MI alone, the new Tables S1, and S2 now summarize the results. As shown in these tables, the GLM does offer improvements over MI alone in terms of %AG and %REDIportal among the predicted editing sites. The MI method alone does not lead to very high %AG values due to existence of sequencing errors in the long reads. (We note that for short reads, the MI step of GIREMI led to >99% A-to-G.) This is the current limitation of third- generation sequencing. We anticipate that with improvements in the sequencing accuracy, MI will be increasingly sufficient and the contribution of GLM will be diminished in the future.

We have added a discussion about the above points in the manuscript (page 10). We also updated the code for L-GIREMI so that the authors can choose to use the MI results alone for data with improved sequencing accuracy in the future.

**Figure R7.** Feature contributions in the GLM. (**a**) Variance explained by each feature (up+down: upstream and downstream nucleotides, ratio: allelic ratio, type: mismatch type). (**b**) %A-to-G among RNA ES predicted by the model after removing one feature.

(**c**) Similar as (b), but for %REDIportal.

# Reviewer #2:

The Authors present a new methodology, called L-GIREMI, able to detect RNA editing events through PacBio long reads. This method is able to handle sequencing errors, typical of third generation sequencers, and to prioritise bona fide editing events as they do not show the significant linkage expected from allelic variants.

The proposed methodology, although not completely original

(see https://doi.org/10.1093/bioinformatics/btx473) seems reasonable and effective and the conclusion adequately supported by the data.

However, I have two major issues that should be adequately addressed.

We appreciate the recognition by the reviewer that our method is reasonable and effective. We wish to clarify the differences between L-GIREMI and the method (SPRINT) presented in the paper mentioned above. L-GIREMI utilizes mutual information between nucleotide variants in the reads and a generalized linear model to identify RNA editing sites, whereas SPRINT analyzes the clustering patterns of SNV duplets. Thus, although both tackling relationships of SNVs in the reads, the two methodologies are fundamentally different.

1) Most of editing events have a low rate affecting a small fraction of transcripts. The use of long reads, which generally provide a lower coverage with respect to short reads may be problematic in some cases. The Authors should provide an accurate evaluation of the sensitivity of the method with robust information on the correlation between observed coverage and detectable editing level.

We appreciate this comment and agree that read coverage strongly limits the sensitivity in capturing RNA editing sites, a limitation that is general to all methods. As shown in Fig. 2e of the manuscript, we observed a decrease in the number of identified editing sites as we down-sampled the reads used for the identification. Here, if sensitivity is evaluated using the raw number of detected editing sites, then the sensitivity will naturally be lower given lower total read coverage. This conclusion is ubiquitous to all methods.

To further address the reviewer’s question, we calculated the sensitivity of L-GIREMI in detecting “identifiable” known editing sites given different total read coverage. We defined identifiable known editing sites as REDIportal sites (a comprehensive database of known RNA editing sites) that were covered by at least 6 long reads in a down- sampled dataset (after passing the pre-filtering procedures described in our Methods). Thus, this definition of sensitivity has a pre-requisite that the sites are identifiable in the first place, avoiding the inclusion of sites that had 0 or low coverage. As shown in Fig. R8 below, the sensitivity of L-GIREMI increases given higher read coverage and it plateaus at about 0.86 given 2.6 million reads.

In general, the coverage of long-read RNA-seq is much lower than short-read RNA-seq, due to the high cost of third-generation sequencing. A comparison of results from short- and long-read RNA-seq datasets is presented below (response to comment #2). As

detailed below, we don’t intend to advocate that long-read RNA-seq should replace short-read for RNA editing identification. Instead, our goal is to present a new method that leverages long-read RNA-seq data for RNA editing analysis because there is a need for such a method given the increasing amount of long-read data becoming available.

We have included this figure as Fig. S2c and updated the manuscript accordingly (page 6).

**Figure R8.** Sensitivity (defined in the text above) of L-GIREMI for down-sampled AD dataset.

2) As this methods is alternative to the use of short reads, the Authors should provide a comparative assessment by analysing the results obtained from both on the same samples.

To compare to the results of short reads, we obtained short-read RNA-seq data of GM12878 (the same cell line used for long-read data in this study) from the ENCODE consortium (cytosolic polyA+ RNA, accession number ENCSR000COR). We identified RNA editing sites using the “genome-aware” method which is a standard method for datasets that have genome information (which is true for GM12878) (PMID: 25730491). All the pre-filters used as part of L-GIREMI were also used here for the short reads data, to enable a fair comparison. In addition, since the short-read RNA-seq data had deep coverage (with a total of >200M reads), we randomly down-sampled the data to retain 30M reads, which yields approximately the same level of base coverage as the long-read data (considering read length differences). Using this down-sampled dataset, this method identified more than 4000 editing sites (86% being A-to-G) in the short reads (new Fig. S5a). Compared to the long-read results (new Table S2), short-read RNA-seq appears to yield higher sensitivity. However, the %A-to-G based on short-read data is relatively low, especially in non-Alu regions (which is partly due to the relatively low total read coverage, known to affect %A-to-G as presented in our previous work (PMID: 23598527)).

We observed that a larger fraction of editing sites from short reads is located in introns, compared to long reads (new Fig. S5b). This may explain the higher number of editing

sites detected in short reads as it is known that introns are enriched with Alu elements where the majority of editing sites reside. To conduct further comparisons, we obtained the union of mismatch sites that were testable (with ≥6 total reads) in both datasets. A total of 190 editing sites were identified in both, with 339 unique to the long reads and 148 unique to the short reads (new Fig. S5c). Sites unique to one dataset were absent in the other dataset mostly due to lack of edited reads. The %A-to-G of the subsets of sites are high, reflecting high quality predictions. Thus, although less editing sites were identified in the long reads, the two modalities perform similarly at common testable sites.

We have included the comparison between long and short-read data on page 7 of the manuscript.

We wish to point out that long-read RNA-seq is not yet a good replacement of short read RNA-seq, given its relatively high cost and high error rate. However, an increasing amount of long-read RNA-seq data is becoming available, which makes it necessary to have appropriate methods that leverage such data for RNA editing analysis. Our method L-GIREMI is, to our knowledge, the first approach to identify RNA editing in long-read RNA-seq data, overcoming the significant challenge of high error rate.

Additionally, they could eventually discuss the use of combined information.

We appreciate this suggestion and have discussed this in the manuscript (page 10).

# Reviewer #3:

L-GIREMI is a new method to detect RNA-editing in the PacBio long-read RNA-seq data using the error profile. L-GIREMI can distinguish the RNA editing sites with mismatches arising from sequencing errors. 98.1% of RNA editing sites identified by the method are A-to-G (Figure 2a), compared to the small fraction of A-to-G of the raw mismatches (Figure 2c). The A-to-G fraction among the predicted RNA editing sites remains relatively high with lower read coverage, although the number of predicted sites decreased. It shows the accuracy of their method is robust for a wide range of total read coverage. In addition to method development, the authors also used the single molecule information plus long read length to test whether the RNA-editing events will co-occur in the same RNA molecule and to conduct the allele-specific RNA editing events analysis. They found a region-skipping pattern of long reads (Figure 5) in the vicinity of RNA editing sites. Below are a few comments to this version of the manuscript:

1. Similar to the authors' previous method GIREMI (Zhang & Xiao,2015), L-GIREMI utilized mutual information to predict RNA editing sites in the first step, given the assumption that most RNA editing events are expected to occur independently of the allelic origin of the mRNA. These predicted RNA editing sites are then used as the training data for the generalized linear model. I wonder if other training data, such as gold standard by experiments, is possible/available to improve the accuracy of

prediction. If it is technically hard to generate such data, but a discussion could be added to explain the limitation of training and possible gaps and improvement.

We appreciate this comment. The quality of training data is critical to the accuracy of predictions. However, it is very challenging to generate enough experimental data to train the model. Currently, L-GIREMI uses the editing sites predicted via mutual information (MI) to train the GLM. In this way, model training is dataset-specific and the user does not need to define or provide any training data. This advantage will be lost if we require experimentally verified gold standard to train the model, as it is hard for each user to generate their own training data, and, alternatively, using a ubiquitous set of training data makes it not data-specific.

We do acknowledge that there are limitations in our strategy, the most significant being its close dependence on the quality of MI-predicted editing sites. As we now show (response to Reviewer 1’s comments), the MI-predicted editing sites typically have

a %AG around 70-80%. In contrast to the short-read method (GIREMI) which had

a %AG >99% among the MI-predicted editing sites, this lower %AG is largely due to existence of sequencing errors in the long-read, which is a current limitation of third- generation sequencing. Therefore, the accuracy of the training data will likely affect the performance of L-GIREMI. However, third-generation sequencing technologies are improving rapidly, we expect this limitation to be mitigated soon with improved sequencing accuracy in the long reads. We have included the above discussion in the revised manuscript (page 10).

2. To further demonstrate L-GIREMI's performance, comparison with other software, including the short-read ones or experimental validation should be added.

Since this is the first method to identify RNA editing sites for long-read RNA-seq, we could not compare to other software of similar purpose. Nonetheless, also responding to Reviewer 1’s comments, we now included comparison to the “genome-aware” method. This is the most intuitive method used for RNA editing identification, that is, to remove genetic variants (if known for the specific sample) or dbSNPs from the predicted RNA editing sites (in conjunction with pre-filtering steps similarly as used for L-GIREMI). We have now included the comparison results as Tables S1 and S2. L-GIREMI outperforms the “genome-aware” method as evaluated by %AG and %REDIportal (% of predicted editing sites that are cataloged as known editing sites in the REDIportal database).

Note that the limited performance of the “genome-aware” method is likely due to high sequencing error rates in the long reads. For details, please refer to our response to Reviewer 1’s comment #3.

In addition, we compared the RNA editing sites identified using short reads and long reads of the same ENCODE cell line GM12878. For details, please refer to our response to Reviewer 2’s comment #2.

3. In addition to the evaluation metric "A-to-G fraction of RNA editing sites", more statistics should be added to evaluate the performance.

Thank you for this comment. We now included more detailed evaluations of the method (also as part of response to Reviewer 1’s comments). Specifically, we have

calculated %REDIportal to compare to known RNA editing sites. We have included evaluation of the predicted editing sites in different types of regions (Alu, non-Alu repeats, and non-repeats which were further separated into coding, introns and UTRs etc). In addition, we have compared our method to the “genome-aware” methods, the performance of which was evaluated via %AG and %REDIportal in different types of regions. As detailed above, we have also compared our results to short-read based results for GM12878.

**Second round of review**

**Reviewer 1**

I thank the authors for the detailed reply. I believe most of my answers were satisfactorily answered. However I still have one major and two minor comments.

First, I am still not convinced by the use of a GLM that selects sites based on the mismatch type. I would like to elaborate on this point since it is, in my opinion, a major issue.

If I understand correctly, the methods proposed in this study works as follows: first, L-GIREMI is applied to produce a set of putative editing sites. The results of this step are not very accurate but nevertheless are highly enriched in true editing sites (as attested by the %A-to-G sites). Then the GLM leverages on this enrichment and uses this set as a learning set to train a GLM that (when combined with L_GIREMI) allows for a substantial improvement of the accuracy as measured by the %A-to-G.

However, as explained by the authors in their reply, actually the main feature used by the GLM is the type of mismatch. If one does not take this feature into account, the GLM actually perform worse than stand-alone L-GIREMI (e.g. %AG decreases from 83% to only 75% for the AD sample). This means that what the GLM does is to "learn" that the learning set is enriched in A-to-G mismatches and then to select sites mostly based onthe fact that they are A-to-G sites (the other features used by the GLM are much less important for classification, as the authors report).

Now, I completely agree that formally there is no explicit bias here towards finding A-to-G sites, as this feature is learned from L-GIREMI results and is not imposed on the model as an external arbitrary constraint. However, since L_GIREMI works sufficiently well to produce 83.3% of A-to-G mismatches, it is clear that the GLM learns this obvious feature and then the additional improvement in the %AG cannot be interpreted as an evidence to an increased accuracy.

To make this point more transparent, let's consider an alternative approach to the one presented in this study: similar to the method presented here, one would choose any RNA-editing detection scheme and run it as a first step, to be followed by a *non-linear* model that would use the results of the first step as a learning set. The only requirement from the first-step method is that it would be good enough to make A-to-G the most abundant MM type (not too difficult to achieve, even methods much worse than L-GIREMI fulfil this criterion). The *non-linear* model to be used is very simple: it is pre-set to choose one and exactly one mismatch type (without specifying in advance which type it would be). Clearly, the model will learn to choose A-to-G mismatches and one would obtain from the model a perfect score of 100% A-to-G. Obviously, this does not mean that the model have improved accuracy in any way.

This is exactly the reason why previous searches for RNA editing sites did not employ the a-priori knowledge that editing is manifested as A-to-G mismatches (or the sequence motif, etc.). One wants to keep the detection pipeline indifferent to the MM type, to allow assessment of the accuracy.

To sum up this lengthy argument: in my opinion if the GLM uses the MM type as a feature, one cannot use the MM type as an independent measure to quantify the accuracy of its results. Thus, it is not at all clear to me that the GLM improves the results. As long as the MM type is used as a selection feature, one should quantify the improvement by alternative measures.

Two minor comments:

1. REDIportal is known to be contain many false-positives (see, e.g. Tan et al, Nature 2017). Since the authors have already applied editing detection using (down-sampled) short reads data for the exact same sample, I believe a better comparison set could be produced by applying genome-aware detection to the full short-reads dataset.

2. I didn't understand the answer to my #11. The authors have now added that "Only the
two most frequent alleles at each site were used", but the formula on p.13 still considers all 16 combinations of the two nucleotides as it sums n_i and n_j over N={A,C,G,T}. I suggest you calculate the MI for each pair using only the two most frequent alleles at each site, and then the sum in the formula would be over four terms only (for example, if the mismatch is A-to-G, the nominator in the four terms would be p(AA), p(AG), p(GA), p(GG)).

Please clarify

**Reviewer 3**

The revision addressed most of the concerns while one remains:
In June 2022, a long-read-based RNA-editing detection software Dinopore was published: Direct identification of A-to-I editing sites with nanopore native RNA sequencing. Nature Methods. doi: 10.1038/s41592-022-01513-3. I encourage to compare L-GIREMI with this software.

**Authors’ response to reviewers**

Reviewer 1:

As for the reviewer’s comment regarding the bias in the GLM model, we have included a discussion to clarify the related point as instructed by Dr. Sands (and Reviewer 3).

Other comments (minor):
1. REDIportal is known to be contain many false-positives (see, e.g. Tan et al, Nature 2017). Since the authors have already applied editing detection using (down-sampled) short reads data for the exact same sample, I believe a better comparison set could be produced by applying genome-aware detection to the full short-reads dataset.

Response: The REDIportal database is a widely used resource by the RNA editing community (the database and its original publication were cited >300 times since 2017). Many of these studies used REDIportal sites as known/gold-standard RNA editing sites, based on which they analyzed editing profiles of various sam-ples, studied the evolution of RNA editing, trained predictive models of editing, etc. Thus, we chose to use REDIportal sites as one alternative way of validating our predictions. In this study, a genome-aware detection using short reads was not possible. This is because the long-read dataset with deep enough cov-erage for down-sampling (from Alzheimer’s disease patient) didn’t have genotype data. On the other hand, the dataset with genotype data (from GM12878) did not have deep enough long-read coverage to enable a comprehensive comparison with short reads.

2. I didn't understand the answer to my #11. The authors have now added that "Only the
two most frequent alleles at each site were used", but the formula on p.13 still considers all 16 combina-tions of the two nucleotides as it sums n_i and n_j over N={A,C,G,T}. I suggest you calculate the MI for each pair using only the two most frequent alleles at each site, and then the sum in the formula would be over four terms only (for example, if the mismatch is A-to-G, the nominator in the four terms would be p(AA), p(AG), p(GA), p(GG)).

Response: Indeed, the MI was calculated using the two most frequent alleles. The formula on p13 was written in general terms to reflect the fact that all variant types were initially considered without bias. To clarify this point, we have updated the formula to explicitly include the two most frequent alleles.

Reviewer #3: The revision addressed most of the concerns while one remains:
In June 2022, a long-read-based RNA-editing detection software Dinopore was published: Direct identification of A-to-I editing sites with nanopore native RNA sequencing. Nature Methods. doi: 10.1038/s41592-022-01513-3. I encourage to compare L-GIREMI with this software.

Response: The above method was developed for Nanopore direct RNA sequencing data. It trains a CNN model to distinguish inosine from adenosine and guanosine in the native RNA sequencing data of Nanopore. Thus, the model is only applicable to Nanopore direct RNA sequencing data. In contrast, L-GIREMI aims to analyze cDNA data (instead of direct RNA). Thus, the two methods are not comparable as they cannot be applied to the same dataset. We appreciate the reviewer’s comment and have added a sentence in the Discussion about Dinopore.
